# Supplementary material for: Monolithically integrated micro-supercapacitors with high areal number density produced by surface adhesive-directed electrolyte assembly
Source: Nat Commun. 2024 Apr 2;15:2850. doi: 10.1038/s41467-024-47216-5 (PMC10987489; doi:10.1038/s41467-024-47216-5)
Supplement: Supplementary file 1 — Supplementary Information [file 41467_2024_47216_MOESM1_ESM.pdf]

Supplementary Information

Monolithically integrated micro-supercapacitors with high areal number density produced by surface adhesive-directed electrolyte assembly

Sen Wang[1], Shuanghao Zheng[1], Xiaoyu Shi[1], Pratteek Das[1], Linmei Li[2], Yuanyuan Zhu[1], Yao Lu[2*], Xinliang Feng[3,4*], and Zhong-Shuai Wu[1,5,6*]

[1]State Key Laboratory of Catalysis, Dalian Institute of Chemical Physics, Chinese Academy of Sciences, 457 Zhongshan Road, Dalian 116023, China

[2]Department of Biotechnology, Dalian Institute of Chemical Physics, Chinese Academy of Sciences, 457 Zhongshan Road, Dalian 116023, China

[3]Center for Advancing Electronics Dresden (cfaed), Faculty of Chemistry and Food Chemistry, Technische Universität Dresden, Dresden 01062, Germany

[4]Max Planck Institute of Microstructure Physics, Halle (Saale) 06120, Germany

[5]Dalian National Laboratory for Clean Energy, Chinese Academy of Sciences, 457 Zhongshan Road, Dalian 116023, China

[6]University of Chinese Academy of Sciences, 19 A Yuquan Road, Shijingshan District, Beijing 100049, China

[*]E-mail: luyao@dicp.ac.cn; xinliang.feng@tu-dresden.de; wuzs@dicp.ac.cn

Table of contents

Supplementary Note 1: Electrochemical characterization of MSC and MIMSCs. 1

Supplementary Fig. 1-18 3

References 21

Supplementary Note 1: Electrochemical characterization of MSC and MIMSCs.

The electrochemical performance of the single MSC was carried out by GCD measurements from 0.15 to 3.85 mA cm^{-2} on an electrochemical workstation (CHI 760E). The electrochemical performance of high-voltage MIMSCs was carried out by CV curves (scanning step from 0.1 to 2 V, test delay of 0.005 s), and GCD profiles from 0.6 to 5 μA on Keithley 2450.

Capacitance values were calculated based on GCD profiles, in term of the following equation:[1]

$$C_{cell} = \frac{It}{\Delta V} \quad (1)$$

$$C_{electrode} = 2C_{cell} \quad (2)$$

Where C_{cell} is the capacitance based on the cell, $C_{electrode}$ is the capacitance contribution mainly from MXene microelectrodes, I is the discharge current from GCD profiles (A), t is the discharge time (second). ΔV is the discharge voltage window (V).

Specific capacitance was calculated in term of the area or volume of the cell according to the following formula:

$$C_A = \frac{C}{A} \quad (3)$$

$$C_V = \frac{C}{V} \quad (4)$$

Where C_A (mF cm^{-2}) and C_V (F cm^{-3}) refers to the areal capacitance and volumetric capacitance, respectively. $A_{electrode}$ and $V_{electrode}$ is the total area (cm^2) and volume (cm^3) of the microelectrodes, respectively. A_{cell} is the entire projected area of the cell, including the area of microelectrodes and the interspaces between them. V_{cell} was calculated by taking into account of the whole volume of the cell, including the volume of MXene microelectrodes, interspaces between the microelectrodes and microcells.

The energy density of the cell was obtained from the equation:

$$E = \frac{1}{2} \times C_{V,device} \times \frac{\Delta V^2}{3600} \quad (5)$$

where E is the energy density (Wh cm^{-3}), $C_{V,device}$ is the volumetric capacitance obtained from equation (4) and ΔV is the discharge voltage (V).

The power density of the cell was calculated from the formula:

$$P = \frac{E}{\Delta t} \times 3600 \qquad (6)$$

where *P* is the power density (W cm^{-3}), and Δt is the discharge time (second).

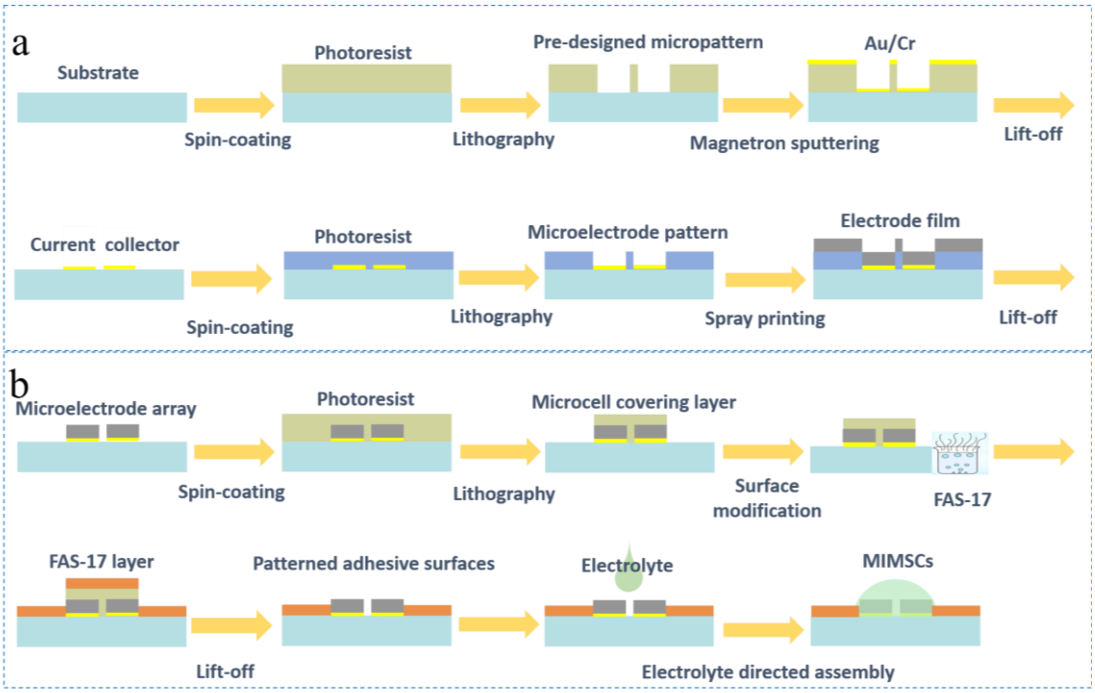

Supplementary Fig. 1 | The detailed cross-section diagrams in fabrication processes of the MIMSCs. (a) Microelectrode arrays of MIMSCs fabricated by multi-step lithographic patterning, magnetron sputtering and spray printing of MXene solution. (b) Fabrication of microelectrode arrays with patterned adhesive surface and localization of electrolyte on the microcell arrays by electrolyte directed assembly strategy.

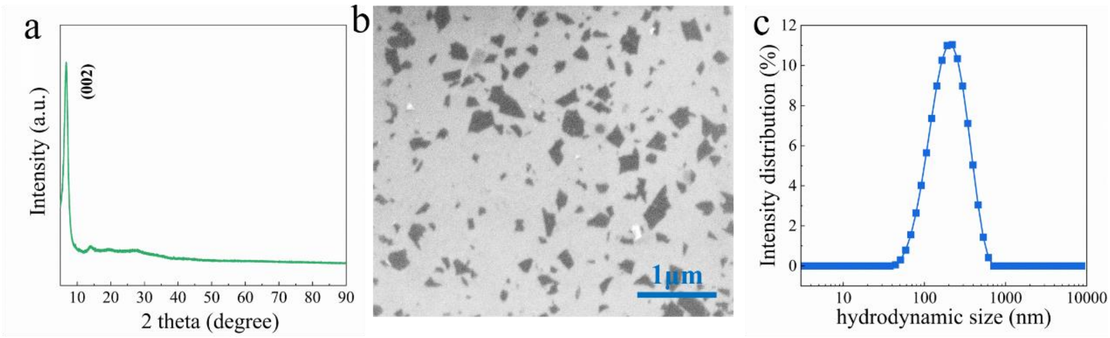

Supplementary Fig. 2 | Characterization of nanometer-sized MXene nanosheets. (a) XRD pattern, (b) SEM image, and (c) dynamic light scattering intensity distribution.

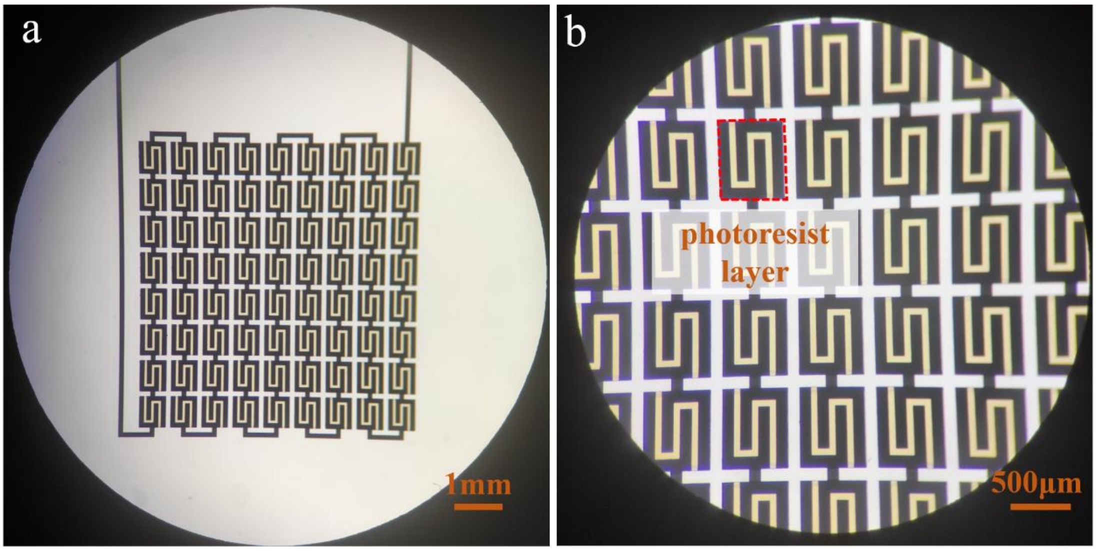

Supplementary Fig. 3 | Optical microscope photographs of microelectrode arrays protected by photoresist covering layer. (a) Low magnification optical microscope photograph, and (b) local magnification optical microscope photograph.

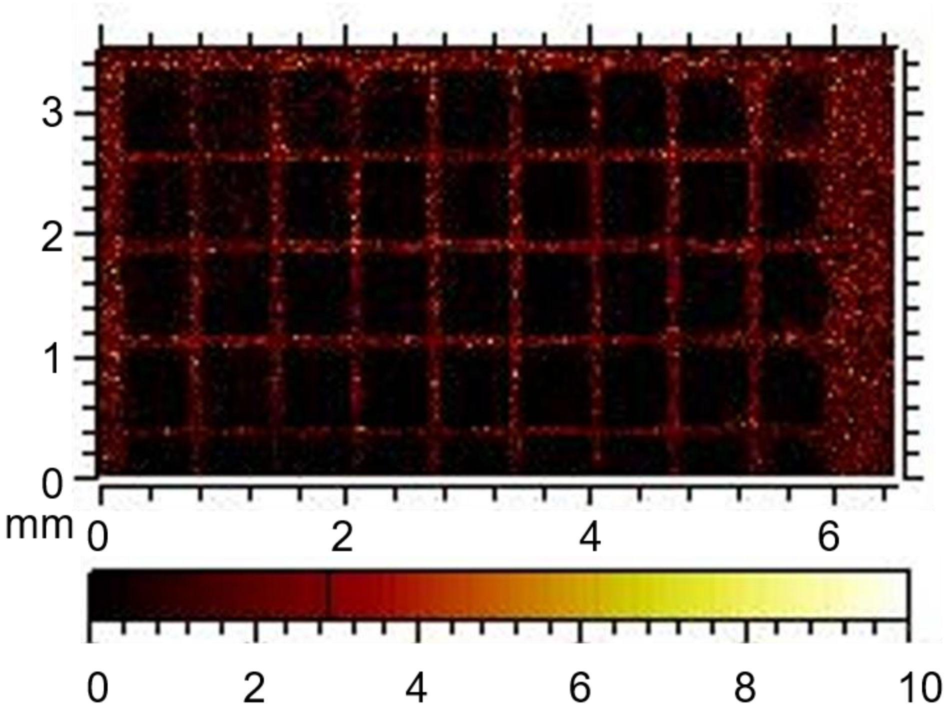

Supplementary Fig. 4 | Consistency characterization of FAS-17 on patterning glass surface. Top view time-of-flight secondary-ion mass spectrometry image of -F fragment of FAS-17 patterning glass surface after lift-off process in ethanol.

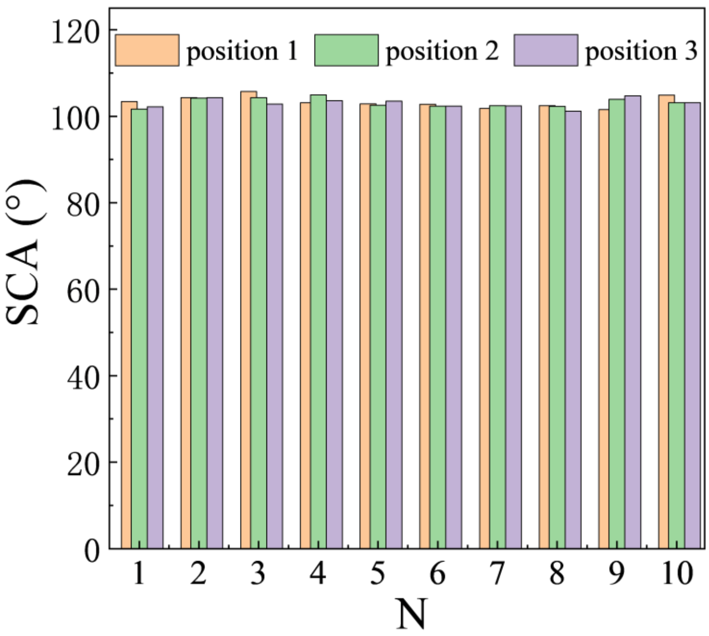

Supplementary Fig. 5 | The stability and consistency of FAS-17 layer on glass during the photoresist lift-off process. The SCAs of water with FAS-17-treated glass at different positions as a function of N, N represents the number of times FAS-17-treated glass soaked in ethanol.

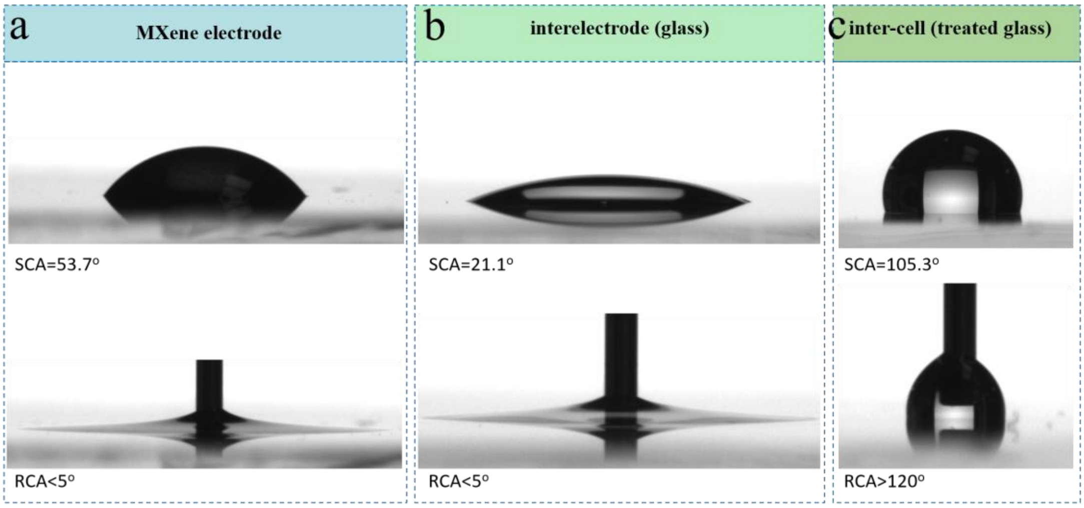

Supplementary Fig. 6 | The wetting behavior between treated MIMSCs with water. The SCA and RCA of water droplets with MXene microelectrode (a), interelectrode glass (b) and inter-cell glass treated with FAS-17 (c).

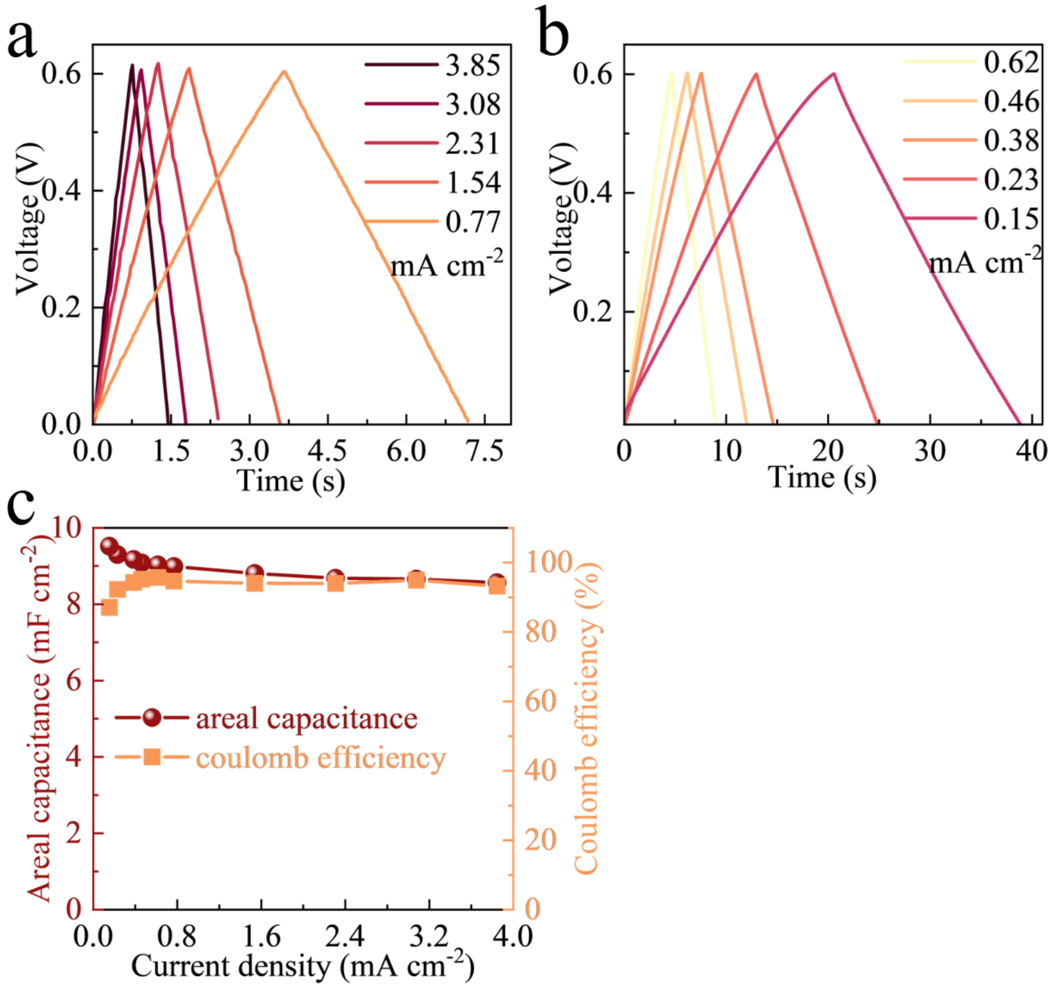

Supplementary Fig. 7 | Electrochemical characterization of a single MSC in mol L^{-1} H_2SO_4 electrolyte. (a,b) GCD profiles over current densities from 0.15 to 3.85 mA cm^{-2}, and (c) areal capacitance and Coulombic efficiency as a function of current density.

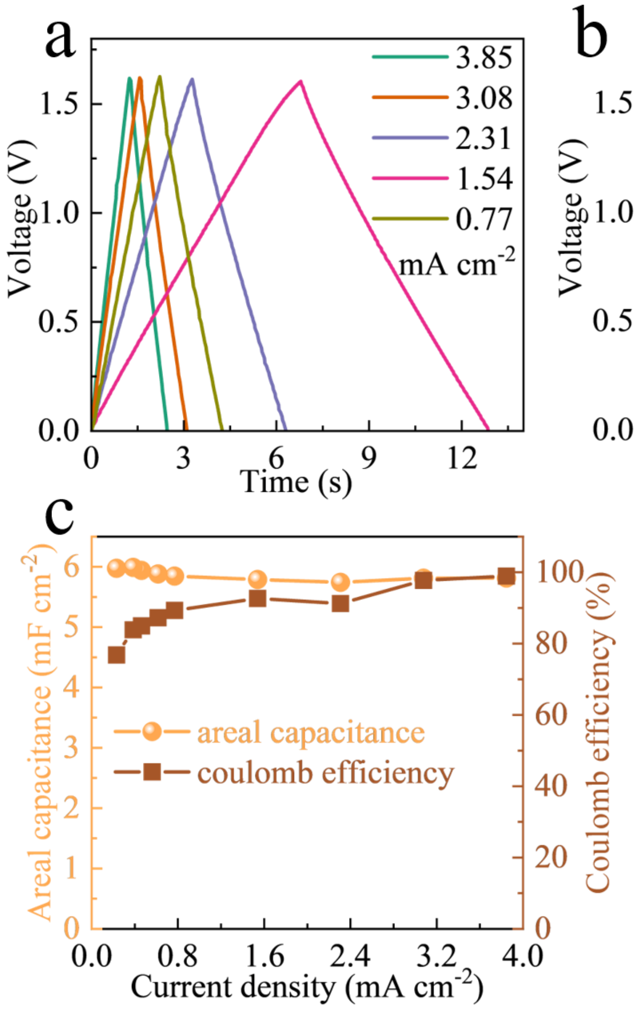

Supplementary Fig. 8 | Electrochemical characterization of a single MSC in 20 mol kg^{-1} LiCl electrolyte. (a,b) GCD profiles over current densities from 0.23 to 3.85 mA cm^{-2}, and (c) areal capacitance and Coulombic efficiency as a function of current density.

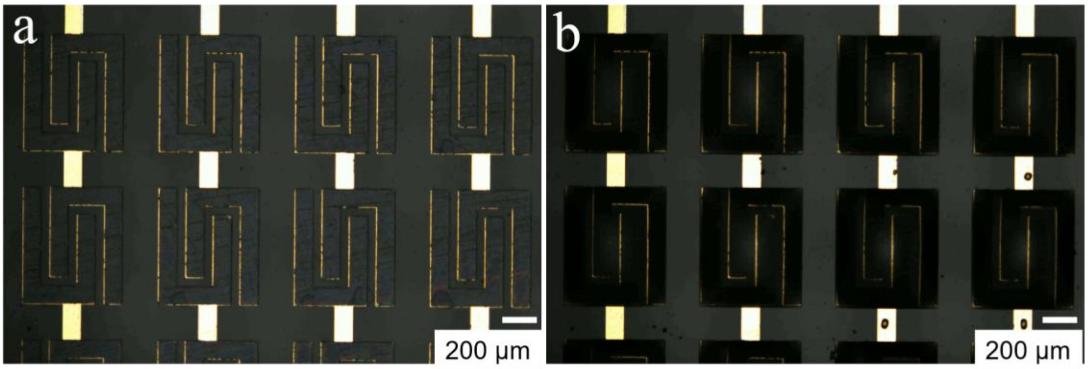

Supplementary Fig. 9 | Microscope images of the spatial control of electrolyte. Microelectrode arrays separated from the adjacent cell with 100 μm without electrolyte (a) and with $EMImBF_4$ electrolyte accurately covering each cell (b).

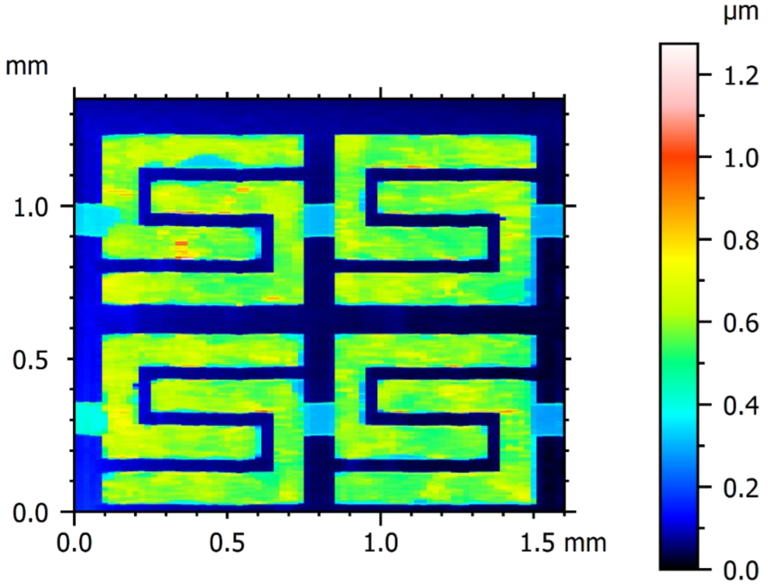

Supplementary Fig. 10 | Thickness consistency characterization of MIMSCs. Thickness mapping of four selected cells in MIMSCs.

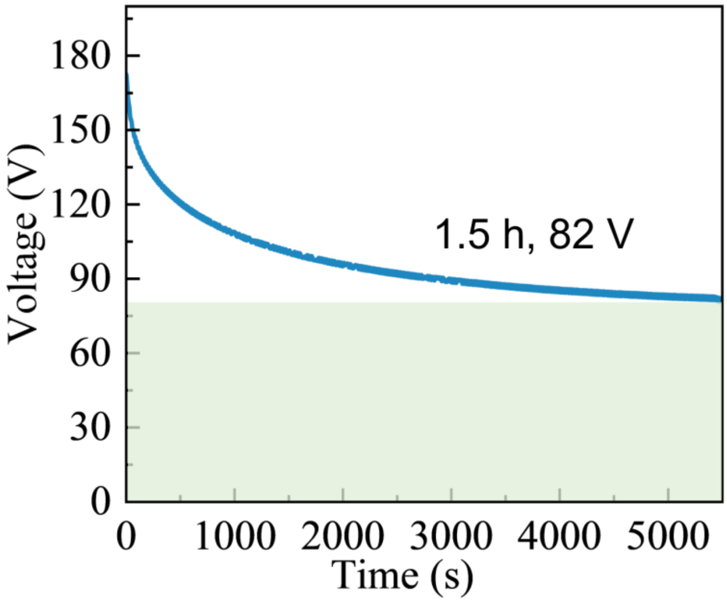

Supplementary Fig. 11 | The self-discharge profile of MIMSCs. It was obtained immediately, after MIMSCs connected 72 microcells in series charging to 190 V. The voltage starting from 180 V is caused by switching the testing software.

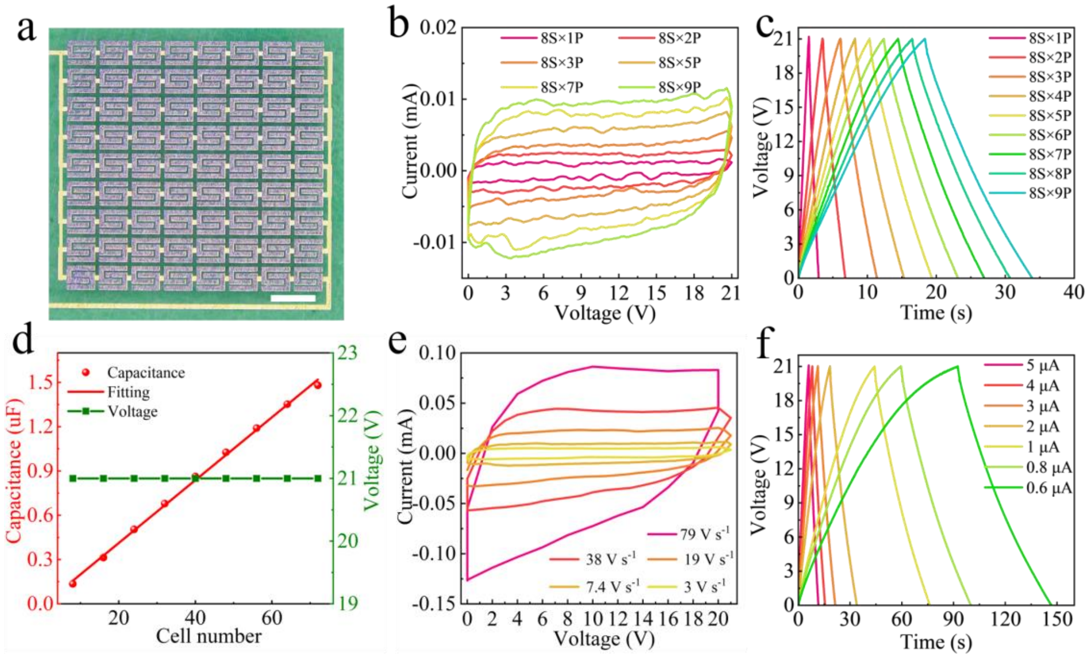

Supplementary Fig. 12 | Tailored capacitance and voltage of MIMSCs in $EMImBF_4$ electrolyte. (a) Photograph of MIMSCs with 72 cells, 8 serially-connected MSCs as a cell pack (8S) and then multiple packs connected in parallel (8S×*y*P, in which y represent the number of cell packs connected in parallel). (b) CV curves obtained at 7.1 V s^{-1}, (c) GCD profiles at 2 μA, and (d) output voltage and capacitance as functions of cell number calculated from GCD profiles of MIMSCs containing 8, 16, 24, 32, 40, 48, 56, 64 and 72 cells. (e,f) CV curves obtained at different scan rates of 3-79 V s^{-1} (e), and GCD profiles at different currents of 0.6-5 μA (f) of MIMSCs containing 72 cells connected in 8S×9P fashion.

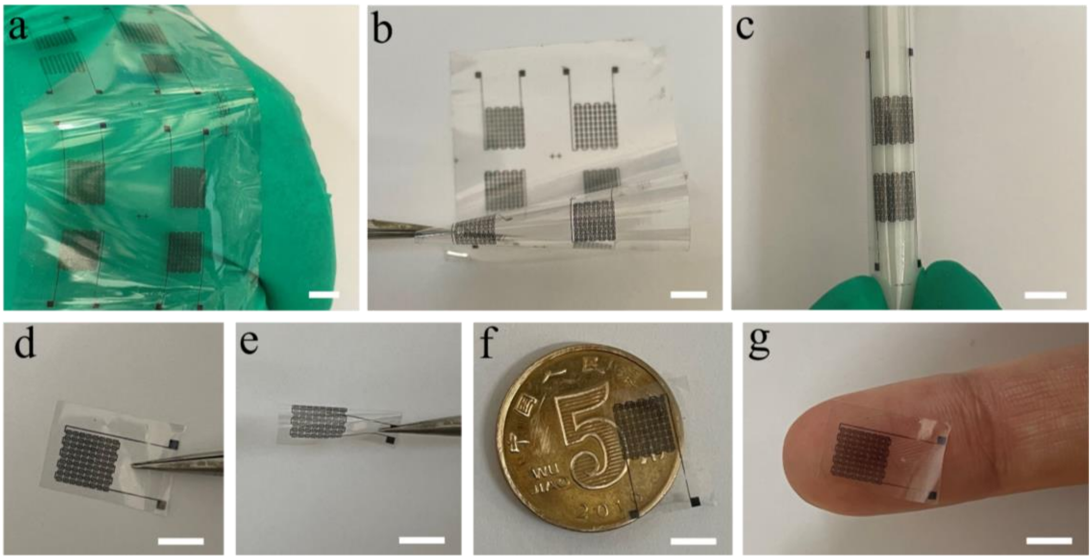

Supplementary Fig. 13 | Flexibility of MIMSCs on a flexible polyethylene terephthalate substrate. (a-g) Optical photographs of flexible MIMSCs with different display forms. All scale bars represent 5 mm.

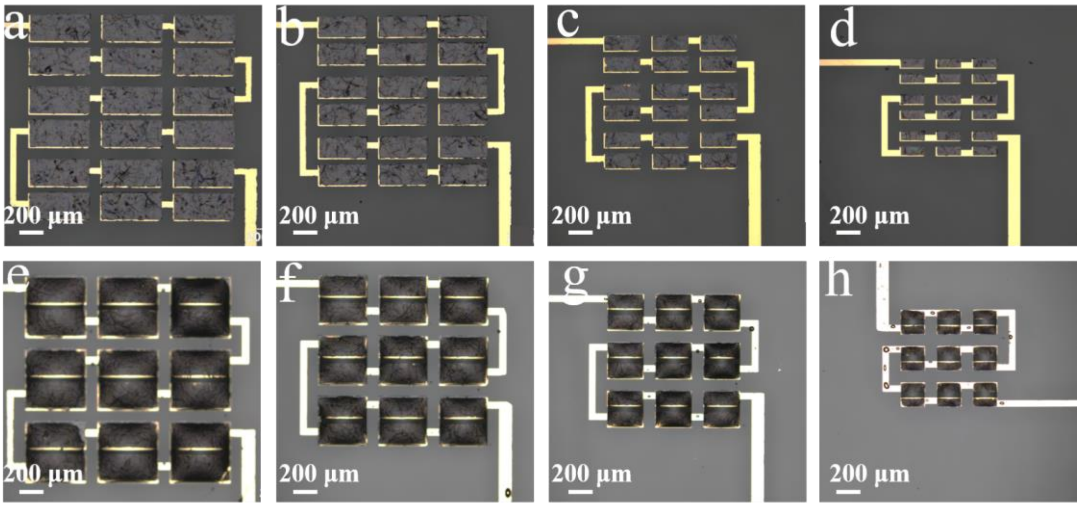

Supplementary Fig. 14 | MIMSCs with higher number density per unit area. Microscope images of MIMSCs with a fixed inter-cell distance (100 μm) and a varying microcell width of 500, 400, 300 and 200 μm, microelectrode arrays without electrolyte (a-d) and with $EMImBF_4$ electrolyte (e-h).

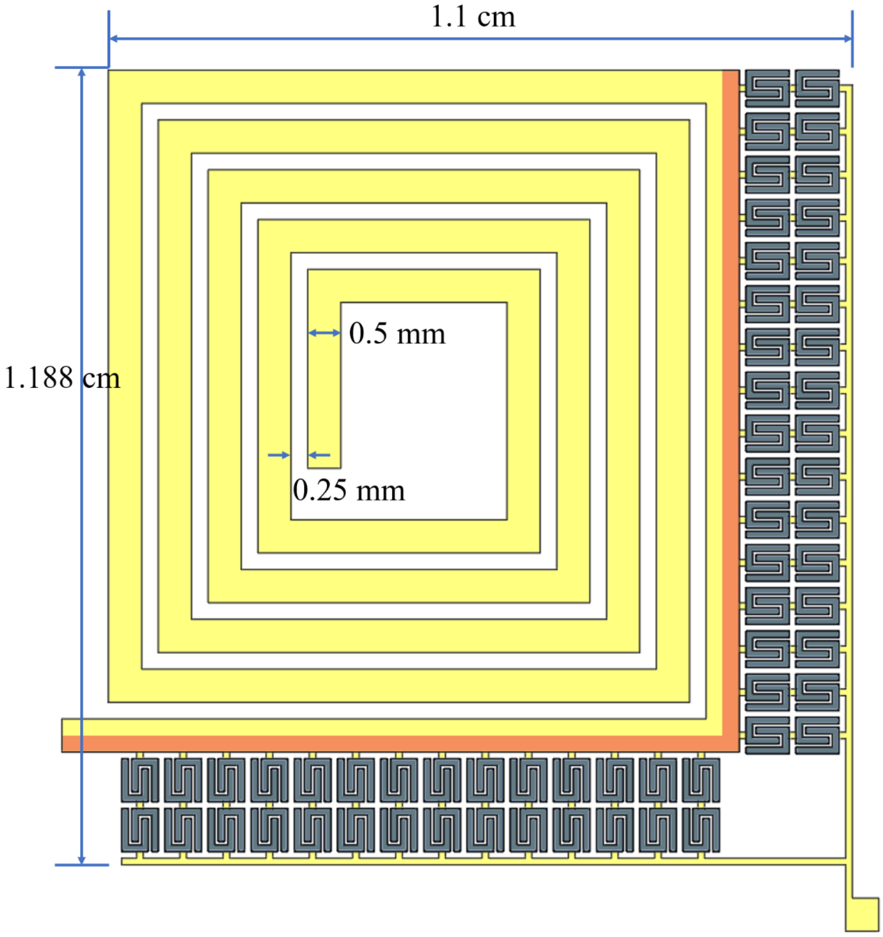

Supplementary Fig. 15 | A dimensional diagram of seamlessly integrated wireless charging MIMSCs microcell. It was drawn using AutoCAD.

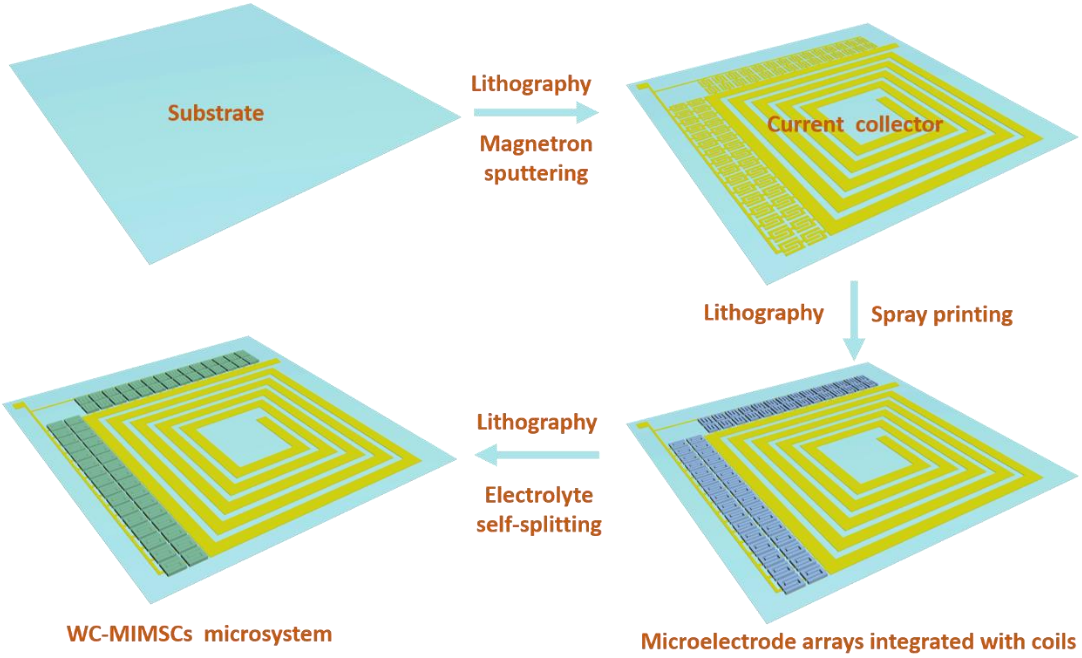

Supplementary Fig. 16 | Schematic of the fabrication of WC-MIMSCs. The seamless coupling of WC-MIMSCs was realized by simply changing the pattern of the lithographic mask during the current collector fabrication process of MIMSCs.

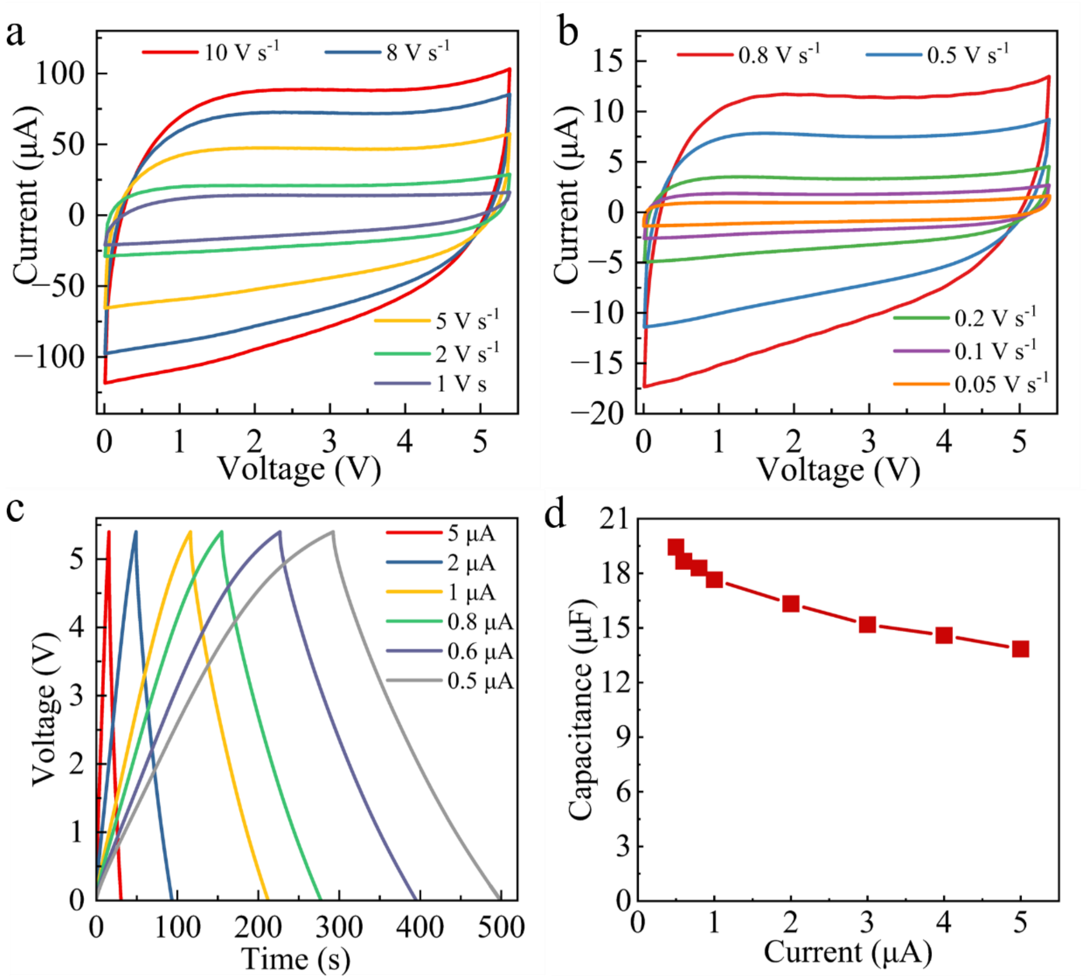

Supplementary Fig. 17 | Electrochemical performance of MIMSCs in WC-MIMSCs in EMImBF$_4$ electrolyte. (a,b) CV curves obtained at different scan rates between 1-10 V s^{-1} (a) , and 0.05-0.8 V s^{-1} (b). (c) GCD profiles at different currents of 0.5-5 μA. (d) Areal capacitance as a function of current.

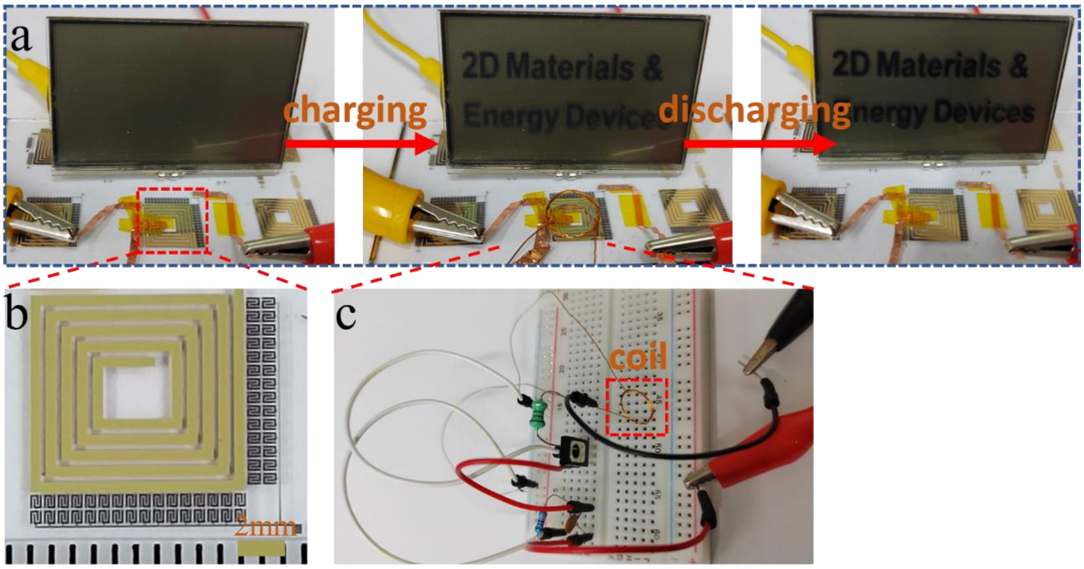

Supplementary Fig. 18 | Optical images of WC-MIMSCs microsystem. (a) Photographs of WC-MIMSCs powering an electronic display screen from charging state to discharging state, the driving voltage is 3 V and the current is less than 5 μA of the display screen used in this study. (b) Enlarged optical photograph of WC-MIMSCs. (c) An optical photograph of the wireless charging transmitter system with a 1 cm circular coil made by copper wire.

Supplementary References

1. Shi, X., et al. Ultrahigh-voltage integrated micro-supercapacitors with designable shapes and superior flexibility. *Energ. Environ. Sci.* **12**, 1534-1541 (2019).
